# Concurrent Validity and Reliability of a Novel Visual Analogue Fitness Perception Scale for Adolescents (FP VAS A)

**DOI:** 10.3390/ijerph18073457

**Published:** 2021-03-26

**Authors:** María Mendoza-Muñoz, José C. Adsuar, David Manuel Mendoza-Muñoz, Patricia Polero, Jorge Carlos-Vivas

**Affiliations:** 1 Health, Economy, Motricity and Education Research Group (HEME), Faculty of Sport Sciences, University of Extremadura, 10003 Cáceres, Spain; mamendozam@unex.es (M.M.-M.); dmendozaj@alumnos.unex.es (D.M.M.-M.); 2 Laboratorio de Biomecánica y Análisis del Movimiento del Litoral, Cenur Litoral Norte, Universidad de la República, Florida 1065, Paysandú 60000, Uruguay; patricia.polero@gmail.com

**Keywords:** adolescence, feasibility, reliable, self-reported physical fitness, self-perception, VAS, youth

## Abstract

Introduction: Self-reported physical fitness (PF) provides an accurate measure of PF, specifically for young people. The Visual Analogue Scale (VAS) is one of the most used psychosocial measurement methods. The main arguments in favor of VAS are its ease of use and comprehension, particularly for less educated participants. There are some scales that assess self-perception of PF, but the VAS presented in this study covers a higher range of responses and a number of variables than other already validated measures. Aims: The aim was to determine the concurrent validity of the Visual Analogue Fitness Perception Scale for Adolescents (FP VAS A) (Sub-study 1) and check its reliability (Sub-study 2). Methods: Anthropometric and body composition measurements were performed, as well as PF tests (manual dynamometry, Course Navette, 4 × 10 m, and sit and reach). The International Fitness Scale (IFIS) and FP VAS A were used to assess self-reported PF. Results: Two sub-studies were carried out: in sub-study 1 a total of 67 students (26 males and 41 females aged 12–16 years) participated. The results showed a significant direct correlation between the level of PF and self-perception of PF (IFIS and FP VAS A), with the FP VAS A obtaining a higher correlation with PF (*r* = 0.444 to 0.666) than the IFIS and PF (*r* = 0.154 to 0.557). In sub-study 2 (test–retest of the FP VAS A), a total of 217 students (120 males and 97 females aged 12–17 years) participated. It showed a moderate reliability for all items; the intraclass correlation coefficient (ICC) was between 0.800 and 0.870, and kappa values ranged from 0.622 (endurance) to 0.458 (flexibility). In addition, Cronbach’s α for the total was 0.860. Conclusion: This study showed good validity and reliability for the FP VAS A in adolescents.

## 1. Introduction

Physical fitness (PF) is defined as the set of physical attributes possessed by an organism for performing different types of physical activity in an efficient and controlled manner [1]. It is determined by genetic factors, environmental factors, and lifestyle, but one’s resulting physical exercise practices are key [2].

Childhood and adolescence are crucial in the development of PF because the lifestyle and healthy behaviors practiced during these early life stages could have an impact on people’s health status and behavior in later life [2].

PF is considered one of the most relevant markers of health [2], and it is a significant predictor of mortality and morbidity due to cardiovascular diseases [3,4]. High levels of PF during childhood and adolescence are inversely related to total and abdominal obesity [5]. Thus, maintaining moderate to high levels of PF during adulthood contributes to reducing the risk of developing chronic diseases prematurely and suffering an early death caused by any of these diseases [6]. Previous studies reported that low levels of PF may constitute a more important risk factor than traditional risk factors such as smoking, obesity, diabetes, hypertension, or high cholesterol [7,8]. In fact, PF is considered to be a powerful health indicator for all ages [7]. This reason leads public health agencies to be interested in assessing PF and investigating different methods of obtaining an effective assessment [2], since PF assessment is relevant for early detection of different pathologies and their prevention.

Measurement methods used to assess PF need to be periodically repeated and sensitive to changes in results. To make any measurement method developed useful and significative, it is necessary to demonstrate its validity (i.e., evidence that it is measuring what it is intended to measure) and reliability (i.e., evidence that the obtained scores are accurate and consistent) [9,10]. In this regard, numerous test batteries have been developed to assess PF in young people and adults, such as the European Physical Fitness (EUROFIT) battery [11], Physical Activity and Health Battery for Adults (AFISAL) [12], test battery for the Assessment of Health-Related Physical Fitness (COFISA) [13], or the Assessing Levels of Physical Activity (ALPHA-Fitness) battery [14]. These tests provide concrete and predictive information about children’s and adolescents’ present and future health. Specifically, the ALPHA-Fitness battery was created with the aim of designing a set of field tests to assess PF with a strong focus on improving health in children and adolescents, establishing its reliability, safety, validity and feasibility [15]. This battery was designed to be globally used by the public health system in the different countries of the European Union. The ALPHA-Fitness battery also offers a manual that allows assignment of a PF level based on every test outcome (ranging from 1 “very low level” to 5 “very high level”) and according to sex and age. Therefore, this battery was chosen for the development of this study due to its reliability, validity, and efficiency for use with adolescents [14].

Physical fitness can be objective and accurate when assessed by laboratory or field tests as specified above. However, it is costly and time-consuming. Thus, the use of these assessments may be slightly limited. Therefore, an alternative method that could be used are self-reported PF surveys, which could provide an accurate measure of self-reported PF with which to identify deficiencies in PF specifically for young people [16].

Specifically, the IFIS was created by Ortega et al. [16] to assess self-perception of PF based on 5 questions in the form of a 5-point Likert scale. However, the Likert scale format has certain limitations in the wording of the descriptive categories, which are likely to affect the subjects’ responses. Moreover, to describe a continuous, complex, and subjective phenomenon, artificial categories may not be enough [17,18,19].

In contrast a Visual Analogue Scale (VAS) is commonly used in psychology research, and it has been suggested as being more responsive than the Likert scale and may be more valid and reliable [17,18]. More specifically, regarding the self-perception of PF, we found that the Self-assessed Physical Fitness scale [19] measures aerobic fitness, muscular strength, endurance, flexibility and balance using a visual analogue scale ranging from poor to good. However, this scale was validated in students in health-related disciplines, which may limit its generalizability to other populations such as adolescents. Moreover, the Self-assessed Physical Fitness scale did not assess the global self-perception of PF. Thus, the development of a VAS for self-perceived physical fitness assessment in adolescents could be interesting.

Therefore, this study aimed to determine the concurrent validity of a novel Visual Analogue Fitness Perception Scale for Adolescents (FP VAS A) (Sub-study 1) and to check the reliability of the FP VAS A (Sub-study 2).

## 2. Materials and Methods

### 2.1. Study Design

A single-measure cross-sectional correlational design was conducted in Sub-study 1, and a two-measure cross-sectional test–retest design was carried out in Sub-study 2.

### 2.2. Ethics Approval

Ethical approval was provided by the Bioethics and Biosafety Committee of the University of Extremadura on 16 March 2020 (approval number: 10/2020), in accordance with the updates of the Declaration of Helsinki, as amended by the 64th General Assembly of the World Medical Association (Fortaleza, Brazil, 2013) and the Law 14/2007, on Biomedical Research.

### 2.3. Sub-Study 1. Concurrent Validity of FP VAS A. Correlations between Self-Perceived PF and Actual PF Level

#### 2.3.1. Sample Calculation

Fifty-three participants were needed to reach a power of 80% to detect a difference of 0.31 between the null hypothesis correlation of 0.29 (very low or close to zero association) and the alternative hypothesis correlation of 0.60 (high association) [20]. The significance level was set at alpha equal to 0.05.

#### 2.3.2. Participants

A total of 67 students from two secondary schools from Extremadura participated in Sub-study 1 (Males: *n* = 26, age = 14.5 (1.6) years, height = 163.2 (12.4) cm, body weight = 51.1 (12.4) kg; Females: *n* = 41, age = 14.8 (1.6), height = 159.2 (6.9) cm, body weight = 52.5 (9.5) kg). All participants met the following inclusion criteria: (1) age between 12 and 18 years; (2) no pathologies contraindicating the participation in PF tests; (3) informed consent signed by parents or legal guardians; (4) participant’s acceptance to participate in the study.

### 2.4. Sub-Study 2. Reliability of the FP VAS A

#### 2.4.1. Sample Calculation

Sixty-three participants with two observations per participant were needed to reach 90% power. The alternative hypothesis was that the intraclass correlation coefficient would be 0.80 (maximum value found in previous studies), and the null hypothesis was that the intraclass correlation coefficient would be below 0.62 (minimum value found in previous studies) [19]. An *F*-test was used with a significance level of 0.05.

#### 2.4.2. Participants

A total of 217 students from four different secondary schools from Extremadura (Spain) participated in Sub-study 2 (Males: *n* = 120, age = 15.5 (2.4) years, height = 166.9 (11.4) cm, body weight = 60.5 (12.8 kg); Females: *n* = 97, age = 15.3 (2.4) years, height = 160.0 (7.5) cm; body weight = 53.8 (9.4) kg). All participants met the following inclusion criteria: (1) age between 12 and 18 years; (2) informed consent signed by parents or legal guardians; (3) participant’s acceptance to participate in the study.

### 2.5. Material and Measurements

Before the first assessment, all participants participated in a familiarization phase for learning about and practicing the different tests and evaluations included in the study. The familiarization phase was conducted one week before the main assessments and consisted of a verbal explanation of every assessment by the evaluator and two trials of every test or measurement.

The following self-perceived PF, anthropometric, and PF measurements and tools were used.

#### 2.5.1. Self-Perceived of PF Measures

- *Fitness Perception Visual Analogue Scale for Adolescents (FP VAS A).* The tool developed consists of a visual analogue scale that evaluates participants’ perception of their own level of fitness based on 5 different items (general fitness status, cardio-respiratory fitness, muscular strength, speed-agility, and flexibility). Every item ranges from 0 “very poor level” to 10 “excellent level”. See Figure S1.
- *The International Fitness Scale (IFIS)* [16] was also used to assess self-perception of PF. This instrument consists of 5 items as a 5-point Likert scale (overall FC, your muscular strength, your cardio-respiratory fitness, your speed-agility, and your flexibility). The response options are “very poor”, “poor”, “acceptable”, “good”, and “very good” (Kappa = 0.45).

#### 2.5.2. Anthropometric Measures

These measurements were performed under standardized conditions, following the protocol established in the Data Collection Procedure Manual developed specifically for the *Childhood Obesity Surveillance Initiative* (COSI) [21]. Before starting any measurement, participants were asked to remove their shoes and socks and any heavy clothes (coats, sweaters, jackets, etc.) or accessory (pockets, belts, etc.). Height was measured with a stadiometer (Tanita Tantois, Tanita Corporation, Tokyo, Japan) placed on a vertical surface with the measurement scale perpendicular to the ground. It was measured with the participant in a standing position, with shoulders balanced and arms relaxed along the body. The outcome was taken in cm, to the nearest mm. Body weight was evaluated using a bioimpedancemeter (Tanita MC-780 MA, Tanita Corporation, Tokyo, Japan) and was recorded in kg, up to the nearest 100 g.

#### 2.5.3. Physical Fitness Measures

The Assessing Levels of Physical Activity (ALPHA-Fitness) battery was applied [14,15,22]. The following indicators were included: (a) upper body strength (manual dynamometry), (b) lower-limb strength (standing long jump); (c) cardiorespiratory aptitude (Course Navette test), and (d) speed-agility (4 × 10 m test). The description of every fitness assessment is described hereunder:

- *Upper-body Strength.* It was measured by manual dynamometry [23,24,25] and using an adjustable digital dynamometer (TKK 5041 Grip D, Takei, Tokio, Japón). The grip was adjusted according to the size of participant’s hand and followed a reference table to set the optimal grip for every participant [25]. During the test, they should squeeze the dynamometer slowly and continuously for 3–5 s. Two trials were alternately performed with both hands. The best result was considered for analysis, recorded in kg to the nearest 0.1 kg. During all administrations, the instrument was kept in line with the forearm with the elbow fully extended, avoiding any contact between the dynamometer and any other body part.
- *Lower-limb Strength.* The standing long jump was used. It was measured in cm, using a PVC tape measure (from the starting line to the point where the back of the heel was closest to the starting line) [26]. Participants started from a standing position behind the starting line with their feet separated at the width of their shoulders. Then, they were instructed to bend their knees, placing their arms in front of their body and parallel to the ground. From this position, they swung their arms, while jumping as far as possible. Two attempts were carried out, recording the best result of them. A third attempt was allowed if participants had not maintained a standing position during the landing phase of jumping.
- *Cardiorespiratory Fitness.* The Course Navette test (20 m shuttle run test) was performed. Participants ran between two lines separated by 20 m, following the rhythm emitted by the audio signals. They started by listening to the audio signal or beep and were instructed to adjust their rhythm to the audio signal and to be at one end of the 20 m track when the player emitted the sound. When they reached one end of the track, they touched the line with their foot, turned sharply, and ran in the opposite direction. The test started at 8.5 km/h, increasing by 0.5 km/h every minute. Participants were encouraged to keep running as long as possible. The test ended when every participant stopped due to fatigue or did not reach the finish line simultaneously with the audio signal or beep on two consecutive occasions [27,28]. The last stage completed by the participant was the score obtained.
- *Speed-Agility*. The 4 × 10 m test was administered [29]. It consisted of covering a total distance of 40 m. Participants had to cover the total distance between two lines separated by 10 m by taking three sponges alternately as fast as possible. They performed two trials, recording the best result in seconds. During the test, participants had to cross the line with both feet in every segment. The test ended when participants crossed the finish line with one foot. The examiner acted as an example and listed the cycles completed by each student.

Additionally, flexibility was also evaluated applying the “sit and reach” test. For this test, a 33 cm high box with a slide rule attached to the top was required. During the test, the subjects had to be seated on the floor and extended both legs fully, with their shoulders apart and their feet placed horizontally against the box. Once this position was adopted, with one hand on top of the other, they slid their hands across the top of the ruler forward until they reached the maximum possible distance and held this position for 2 s for the trial to be valid. The mark was taken in centimetres, taking as a reference the finger that reached the greatest distance [30].

For subsequent analysis, all the physical tests mentioned were weighted according to the sex and age of the subjects, taking the ALPHA-Fitness battery manual as a guide [22,25,29], establishing a score from 1 to 5, where 1 corresponded to a very low level and 5 to an excellent level. In the case of flexibility, the score for this test was evaluated according to age and sex, taking as a reference the percentiles of the HELENA study [31].

### 2.6. Statistical Analysis

All information collected was tabulated in a database designed for this study. Statistical analyses were carried out using SPSS (Version 25, IBM SPSS, Chicago, IL, USA) software and personal data were kept anonymous.

Data are presented as mean and standard deviation and median and interquartile range both for the total sample and segmented by sex.

Normality and homogeneity were tested using the Kolmogorov–Smirnov test and Levene’s test, respectively.

Independent *t*-tests were applied to analyze between-sex differences for parametric variables (height, weight, and FP VAS-FX), and the Mann–Whitney U test was used for nonparametric variables (age, all FP VAS items (except FP VAS-FX), all IFIS items, and all variables in relation to the PF tests). Differences were considered significant at *p* ≤ 0.05.

The internal reliability of the scale was defined by the Cronbach’s coefficient of the total scale. Relative reliability was determined by the intraclass correlation coefficient (ICC_3,1_) [32]. Absolute reliability was determined by the standard error of measurement (SEM) and the minimum real difference (MRD) [33]. The test–retest reliability was conducted with two assessments 15 days apart. Statistical significance was determined by *p* ≤ 0.05. To interpret the ICC, the following classification was used [34]: ICC < 0.50, low reliability; 0.50 to 0.75, moderate; 0.75 to 0.90, good; and >0.9, excellent. In addition, for the test–retest study and to measure the reproducibility of the total responses, Kendall’s tau-b and the kappa index were calculated too. Kendall’s tau-b was determined according to the modified Fleiss criteria [35]: <0.35, poor or inadequate agreement; 0.40–0.75, acceptable agreement; and >0.75, excellent agreement. The kappa values were interpreted as following the classification of Landis and Koch [36]: <0.00, poor; 0.00 to 0.20, light; 0.21 to 0.40, fair; 0.41 to 0.60, moderate; 0.61 to 0.80, substantial; and 0.81 to 1, almost perfect.

Finally, between-variable relationships were analyzed applying Pearson’s (parametric variables) and Spearman’s correlation coefficients (nonparametric variables). The Bonferroni correction was applied based on the formula α* = α/n−1 [37], where α* is the corrected value at which the null hypothesis should be rejected and n is the number of hypothesis pairs. Therefore, the alpha significance level was set at 0.005 for multiple comparisons between the level of PF and self-perception of PF. Correlation values were interpreted following Cohen’s classification [20]: 0.30 to 0.59, moderate correlation; 0.60 to 0.79, high; and ≥0.80, excellent.

## 3. Results

### 3.1. Sub-Study 1. Concurrent Validity of the FP VAS A. Correlations between Self-Perceived PF and Actual PF Level

A total of 67 adolescents participated in Sub-study 1, 26 boys (38.8%) and 41 girls (61.2%). Table 1 shows the characterization of the general sample, segmented by sex, as well as the significant differences between sexes. It shows that FP VAS-CF (*p* = 0.035), FP VAS-S (*p* = 0.021), IFIS values (except for IFIS-FX), and level of flexibility were significantly higher in boys than girls.

A significant association was found between FP VAS A and IFIS with the level of FC (Table 2). Overall, the results showed a significant direct correlation between the level of PF and self-perception of PF. Specifically, FP VAS A showed a higher level of correlation (*r* = 0.444 to 0.666, *p* < 0.001) than the IFIS (*r* = 0.154 to 0.557, *p* = 0.214 to *p* < 0.001).

### 3.2. Sub-study 2. Reliability of FP VAS A

Table 3 shows the participants’ characteristics and the outcomes regarding between-sex comparisons. The total sample size was 217, with a similar number of males and females (55.3% males versus 44.7% females). Results revealed significantly greater values for height (*p* < 0.001) and all FP VAS A items for males compared to females. Additionally, females presented a significantly lower body weight (*p* < 0.001) than males.

Table 4 shows the test–retest reliability statistics in adolescents for the five items that make up the FP VAS A. ICC (between 0.800 and 0.870) and kappa values (between 0.622 to 0.458) showed a moderate reliability for all items. Furthermore, the Cronbach’s α for the total was 0.860. In addition, SEM% oscillated between 7.1% to 17.3%, but almost all of the items were around 10%. SRD% oscillated between 19.6% to 47.8%, but the rest of the items were around 29%.

## 4. Discussion

This study explored the specific correlations between self-reported PF and real PF level for establishing the concurrent validity of FP VAS A and examining its reliability.

The main findings of this study determined a good concurrent validity between the PF and the FP VAS A (*r* = 0.444 to 0.666), as well as a good reliability for the FP VAS A (ICC = 0.800 and 0.870). Moreover, a higher correlation was found between participants’ PF level and the FP VAS A than between PF and the IFIS.

Regarding the relationships obtained between participants’ PF level and their self-perception of PF, our results were in accordance with previous studies that showed these correlations in different populations, such as children [38], young people [16], and adults [39,40]. Specifically in adolescents, our study shows that there was a moderate correlation between objectively measured PF level and self-reported PF, as measured by the FP VAS A (*r* = 0.444 to 0.666), as well as with the IFIS (*r* = 0.410 to 0.557), with the exception of strength level. Along this line, Ortega et al. [16] reported similar results in this same population (*r* = 0.540 to 0.650). However, Jürimäe and Saar [41], despite finding an association of self-reported PF with endurance and flexibility, did not find it for strength; this may be due to the fact that their study compared participants in different age groups, the sample was homogeneous, and the sample size was very small (*n* from 56 to 70).

Furthermore, it can be observed that the correlations established with the FP VAS A scale are slightly stronger than the IFIS correlations, so it could be used interchangeably. In general, VAS scales are widely used in the psychosocial field, due to their easy administration and comprehension [42,43,44,45]. Therefore, the FP VAS A scale could be simpler for participants with less training, younger age, or difficulty with comprehension.

The main outcomes of between-sex comparisons showed that males presented better self-perception of PF level compared with females. Similarly, a previous study by Ortega et al. [16] showed that males had a superior self-perception of their level of PF in comparison with their female counterparts, with the exception of flexibility. However, our study failed to find any significant difference between male and female volunteers. This may be due to a substantially lower number of participants recruited for the second study (*n* = 67) than the first (*n* = 217), due to a larger sample size being needed for the latter in order to achieve higher statistical power. 

Regarding the reliability of the FP VAS A, we suggest the it showed good reliability (ICC = 0.80 to 0.87) similar to previous studies that used similar scales, such as the IFIS [46,47] or the VAS Self-assessed Physical Fitness scale (ICC = 0.62 to 0.80) [19]. Specifically, Ortega et al. [16] conducted a similar study to the present one, in which adolescents completed the IFIS scale on two occasions 15 days apart and obtained kappa values between 0.54 (muscular strength) and 0.65 (overall FC), with an average of 0.59, and thus showing similar results to this study, where a kappa between 0.45 (flexibility) and 0.62 (cardiorespiratory endurance) and an average kappa of 0.57 were obtained. Therefore, both can be considered to have moderate agreement [36].

Based on all abovementioned, the present study shows how the developed questionnaire gives a moderately good estimate of self-perceived PF. Therefore, the FP VAS A could be considered a useful and reliable tool to estimate PF in adolescent populations, offering an alternative to the IFIS [16], which only offers five response options. Having too many response options could lead to difficulties in making choices, while having too few response options can leave respondents with insufficient choice or sensitivity. A respondent may therefore be forced to choose an answer that does not reflect his or her true intention. However, the main arguments in favor of VAS are its ease of use and comprehension [43,44]. A VAS has been used by several evaluators who claim that it is easier to use than a Likert scale [17,48]. In relation to self-perception of PF, the VAS Self-assessed Physical Fitness scale [19], could be an interesting alternative to the FP VAS A: nevertheless, the former does not consider general PF. Thus, the validated FP VAS A could provide an alternative to either the IFIS or the Self-assessed Physical Fitness scale. Furthermore, assessing self-perceived PF could be an interesting option for screening the population or for use at times when other appropriate measurements cannot be carried out, such as in situations of confinement, or a lack of time or personal or material resources.

The main limitation of the study is that Sub-study 1 was based on a relatively small sample (*n* = 67), despite meeting the sample calculation to achieve acceptable power. A larger sample size could have provided increased statistical power and the possibility of finding significant differences in self-perceived physical fitness assessed with FP VAS A as in Sub-study 2 (*n* = 217). Furthermore, other factors must be taken into account (age, socio-economic status, parental education, media, etc.) that could influence the self-perception of PF. Thus, more studies would be necessary. In addition, it could be interesting to investigate the relationship between self-perception of physical fitness and aspects influencing mental health, not just its relationship with increased body weight or greater or lesser risk of developing chronic disease. Since perceptions of general physical fitness have been shown to be related to self-reported health status, life satisfaction, and health-related quality of life, it suggests that improving general physical fitness may exert a favorable effect on positive health perceptions [49,50].

## 5. Conclusions

Based on our results, we conclude that the FP VAS A is a valid and reliable tool for the assessment of self-reported physical fitness in adolescents. Thus, this study showed good reproducibility for the FP VAS A, as well as a moderate direct correlation between the level of PF of the participants and their self-perception of it—a stronger level of PF being associated with better values of self-perception of it and vice versa. Both the FP VAS A and the IFIS showed this correlation with PF, being higher for the former, which suggests that both measures could be used interchangeably. Therefore, this study provides a tool for the assessment of self-perception of PF as an indirect measure of PF. Its advantage is that it could be used to assess self-perception of PF in large groups in a short period of time, thereby saving the time and resources of researchers.

## Figures and Tables

**Table 1:** Participants’ anthropometry, FP VAS A and IFIS values and level of PF.

		Total	Boys	Girls	*p*
*n* (%)		67 (100)	26 (38.8)	41 (61.2)	
Age (years)	Mean (SD)	14.64 (1.58)	14.46 (1.58)	14.75 (1.59)	0.431
	Median (IR)	14.00 (2.00)	14.00 (2.00)	14.00 (2.00)	
Height (cm)	Mean (SD)	160.77 (9.51)	163.19 (12.41)	159.24 (6.85)	0.069
	Median (IR)	160.00 (12.00)	165.00 (20.25)	159.00 (8.5)	
Body weight (kg)	Mean (SD)	51.95 (10.64)	51.07 (12.37)	52.51 (9.54)	0.545
	Median (IR)	52.00 (12.90)	49.75 (20.88)	52.40 (11.2)	
BMI (kg/m^2^)	Mean (SD)	20.00 (3.28)	18.98 (3.22)	20.65 (3.18)	0.009
	Median (IR)	19.22 (4.13)	17.76 (2.62)	20.47 (3.98)	
**FP VAS**					
FP VAS-GPF	Mean (SD)	8.88 (3.30)	7.18 (1.82)	6.68 (1.52)	0.089
	Median (IR)	8.00 (2.00)	8.00 (1.50)	7.00 (2.00)	
FP VAS-CF	Mean (SD)	6.50 (1.90)	6.96 (2.06)	6.22 (1.76)	0.035
	Median (IR)	8.00 (2.00)	8.00 (2.00)	6.00 (3.00)	
FP VAS-MS	Mean (SD)	6.30 (1.56)	6.62 (1.48)	6.08 (1.58)	0.265
	Median (IR)	6.00 (2.00)	7.00 (2.00)	6.00 (2.00)	
FP VAS-S	Mean (SD)	7.30 (1.60)	7.18 (1.56)	6.80 (1.54)	0.021
	Median (IR)	8.00 (2.00)	8.00 (2.00)	7.00 (2.00)	
FP VAS-FX	Mean (SD)	5.84 (1.90)	6.30 (2.02)	5.56 (1.76)	0.062
	Median (IR)	6.00 (2.00)	6.50 (3.00)	5.00 (2.00)	
**IFIS**					
IFIS-GPF	Mean (SD)	3.80 (0.78)	4.11 (0.81)	3.61(0.70)	0.008
	Median (IR)	4.00 (1.00)	4.00 (1.00)	4.00 (1.00)	
IFIS-CF	Mean (SD)	3.45 (0.82)	3.73 (0.87)	3.32 (0.74)	0.015
	Median (IR)	3.00 (1.00)	4.00 (1.00)	3.00 (1.00)	
IFIS-MS	Mean (SD)	3.37 (0.71)	3.57 (0.85)	3.24 (0.58)	0.046
	Median (IR)	3.00 (1.00)	4.00 (1.00)	3.00 (1.00)	
IFIS-S	Mean (SD)	3.76 (0.87)	4.07 (0.89)	3.56 (0.80)	0.018
	Median (IR)	4.00 (1.00)	4.00 (2.00)	4.00 (1.00)	
IFIS-FX	Mean (SD)	3.00 (0.83)	3.07 (0.79)	2.95 (0.86)	0.745
	Median (IR)	3.00 (0)	3.00(0)	3.00 (1.00)	
**PF test**					
GPF	Mean (SD)	3.07 (0.84)	3.21 (0.96)	2.99 (0.75)	0.397
	Median (IR)	3.25 (1.25)	3.25 (1.62)	3.00 (1.00)	
CF	Mean (SD)	3.69 (1.34)	3.46 (1.33)	3.82 (1.34)	0.235
	Median (IR)	4.00 (2.00)	3.50 (3.00)	4.00 (2.00)	
MS	Mean (SD)	2.30 (1.39)	2.15 (1.43)	2.39 (1.37)	0.372
	Median (IR)	2.00 (2.00)	1.50 (2.00)	2.00 (2.00)	
S	Mean (SD)	3.43 (1.54)	3.50 (1.65)	3.39 (1.48)	0.704
	Median (IR)	4.00 (3.00)	4.00 (3.25)	4.00 (3.00)	
FX	Mean (SD)	2.89 (1.39)	3.73 (1.28)	2.36 (1.19	<0.001
	Median (IR)	2.00 (2.00)	4.00 (2.00)	2.00 (1.00)	

FP VAS A: Fitness Perception Visual Analogue Scale for adolescents. IFIS: International Fitness Scale. PF: physical fitness. GPF: general physical fitness; CF: cardiorespiratory fitness; MS: muscular strength; S: Speed; FX: flexibility. Data are shown as mean (SD) and median (IR). Alpha level was set at *p* ≤ 0.05.

**Table 2:** Relationship between FP VAS A and IFIS with the level of PF.

	Physical Fitness Level				
	GPF	CF	MS	S	FX
**FP VAS** **A—GPF**	0.595 **				
**IFIS—GPF**	0.465 **				
**FP VAS A—CF**		0.533 **			
**IFIS—CF**		0.410 **			
**FP VAS** **A—MS**			0.577 **		
**IFIS—MS**			0.154		
**FP VAS** **A—S**				0.666 **	
**IFIS—S**				0.557 **	
**FP VAS A—FX**					0.444 **
**IFIS—FX**					0.417 **

FP VAS A: Fitness Perception Visual Analogue Scale for adolescents. IFIS: International Fitness Scale. GPF: general physical fitness; CF: cardiorespiratory fitness; MS: muscular strength; S: Speed; FX: flexibility; Data are presented as *r* value; ** *p* < 0.001.

**Table 3:** Participants’ anthropometry and FP VAS values.

		Total	Male	Female	*p*
*n* (%)		217	120 (55.3)	97 (44.7)	
**Age (years)**	Mean (SD)	15.37 (2.37)	15.47 (2.40)	15.26 (2.35)	0.334
	Median (IR)	14.00 (5.00)	14.00 (4.00)	14.00 (4.50)	
**Height (cm)**	Mean (SD)	163.81 (10.39)	166.87 (11.40)	160.04 (7.47)	<0.001
	Median (IR)	164.00 (14.00)	167.50 (16.5)	160.00 (10.00)	
**Body weight (kg)**	Mean (SD)	55.95 (12.70)	60.48 (12.79)	53.76 (9.44)	0.084
	Median (IR)	54.10 (20.00)	60.00 (10.00)	53.00 (12.00)	
**BMI (kg/m^2^)**	Mean (SD)	20.64 (3.21)	20.41 (3.30)	20.93 (3.09)	0.201
	Median (IR)	20.39 (5.37)	19.95 (6.03)	20.63 (4.24)	
**FP VAS A** **—GPF**	Mean (SD)	7.01 (1.38)	7.32 (1.30)	6.64 (1.38)	<0.001
	Median (IR)	7.00 (2.00)	7.00 (1.00)	7.00 (2.00)	
**FP VAS** **A—CF**	Mean (SD)	6.56 (1.79)	6.87 (1.85)	6.17 (1.63)	0.001
	Median (IR)	7.00 (3.00)	7.00 (2.00)	6.00 (2.00)	
**FP VAS** **A—MS**	Mean (SD)	6.42 (1.56)	6.69 (1.60)	6.08 (1.45)	0.001
	Median (IR)	7.00 (1.00)	7.00 (2.00)	6.00 (2.00)	
**FP VAS** **A—S**	Mean (SD)	7.17 (1.65)	7.72 (1.51)	6.47 (1.55)	<0.001
	Median (IR)	7.00 (2.00)	8.00 (2.00)	7.00 (1.50)	
**FP VAS A** **—FX**	Mean (SD)	5.47 (2.17)	5.56 (2.29)	5.37 (2.02)	0.350
	Median (IR)	5.00 (4.00)	6.00 (5.00)	5,00 (3.00)	

FP VAS A: Fitness Perception Visual Analogue Scale for adolescents (score 0 to 10). BMI: body mass index. GPF: general physical fitness; CF: cardiorespiratory fitness; MS: muscular strength; S: Speed; FX: flexibility. Data are shown as mean (SD) and median (IR). Alpha level was set at *p* ≤ 0.05.

**Table 4:** Test–retest reliability of the FP VAS A in two measurements with an interval of 15 days between measurements.

*n*= 217	Mean (SD)Test (Points)	Mean (SD)Retest (Points)	*p*	Kappa	Tau b	Tau c	ICC	SEM	% SEM	SRD	SRD %
**FP VAS** **A—GPF**	7.01 (1.38)	7.21 (1.41)	<0.001	0.591	0.787	0.685	0.87 (0.83–0.90)	0.50	7.1	1.39	19.6
**FP VAS A** **—CF**	6.56 (1.79)	6.70 (1.72)	0.021	0.622	0.760	0.707	0.84 (0.80–0.88)	0.70	10.6	1.95	29.3
**FP VAS A** **—MS**	6.42 (1.56)	6.41 (1.59)	0.968	0.579	0.729	0.654	0.82 (0.78–0.86)	0.67	10.4	1.85	28.9
**FP VAS** **A—S**	7.17 (1.65)	7.06 (1.77)	0.228	0.601	0.732	0.681	0.80 (0.75–0.85)	0.76	10.7	2.12	29.8
**FP VAS** **A—FX**	5.47 (2.17)	5.31 (1.99)	0.074	0.458	0.689	0.655	0.80 (0.75–0.85)	0.93	17.3	2.58	47.8

FP VAS A: Fitness Perception Visual Analogue Scale for adolescents (score 0 to 10). GPF: general physical fitness; CF: cardiorespiratory fitness; MS: muscular strength; S: Speed; FX: flexibility. Data are shown as mean (SD) and median (IR). Alpha level was set at *p* ≤ 0.05. ICC: intraclass correlation coefficient; SEM: standard error of measurement; SRD: small real difference.

## Data Availability

The datasets used during the current study are available from the corresponding author on reasonable request.

## Supplementary Materials

The following are available online at https://www.mdpi.com/article/10.3390/ijerph18073457/s1, Figure S1: FP VAS A scale.
