# Supplementary material for: Concurrent Validity and Reliability of a Novel Visual Analogue Fitness Perception Scale for Adolescents (FP VAS A)
Source: Int J Environ Res Public Health. 2021 Mar 26;18(7):3457. doi: 10.3390/ijerph18073457 (PMC8037782; doi:10.3390/ijerph18073457)

ANEXO I

Escala Visual Analógica de Percepción de Condición Física para Adolescentes

(EVA PCFA) (Spanish Version).

Nombre y apellidos:

Contesta las preguntas tu solo, no hay respuestas correctas o incorrectas. Se sincero y contesta todas las preguntas.

Por favor, piensa en tu nivel de condición física y elige la opción más adecuada.

1. **Mi forma física global es:**

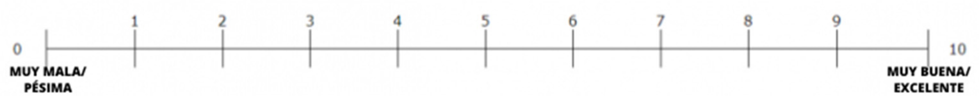

2. **Mi resistencia cardiorrespiratoria** (capacidad para hacer actividades físicas durante mucho tiempo) **es:**

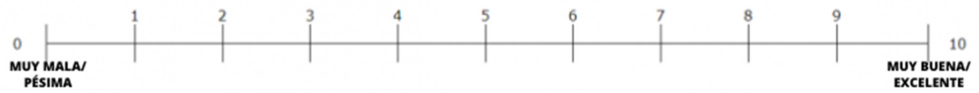

3. **Mi fuerza muscular global es:**

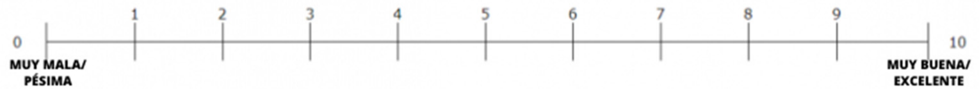

4. **Mi velocidad de desplazamiento** (la capacidad de correr muy rápido) **es:**

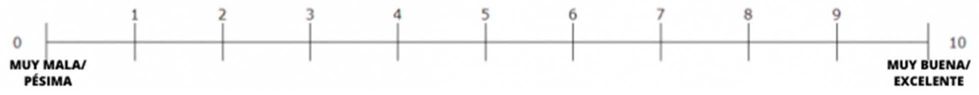

5. **Mi flexibilidad global es:**

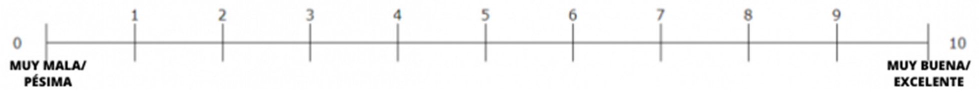

Visual Analogical Scale of Physical Fitness Perception for Adolescents

(VAS PF A) (English Version).

First and last name:

Answer the questions on your own, there are no right or wrong answers. Be honest and answer all the questions.

Please think about your fitness level and choose the most appropriate option.

1. **My overall fitness level is:**

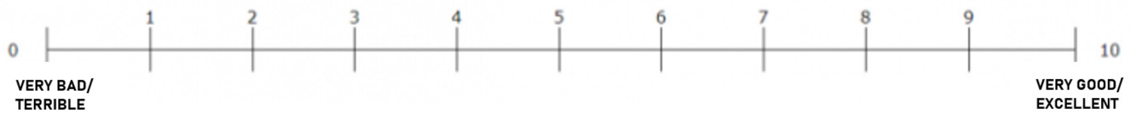

2. **My cardiorespiratory endurance** (ability to do physical activities for a long time) **is:**

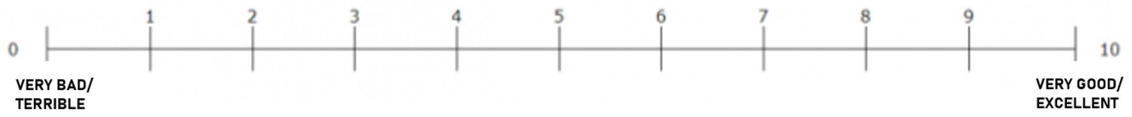

3. **My overall muscular strength is:**

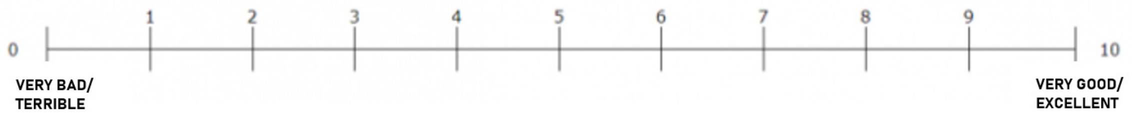

4. **My running speed** (the ability to run very fast) **is:**

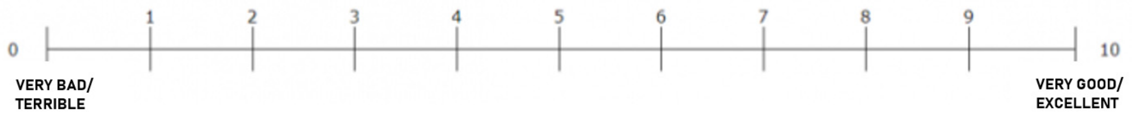

5. **My overall flexibility is:**

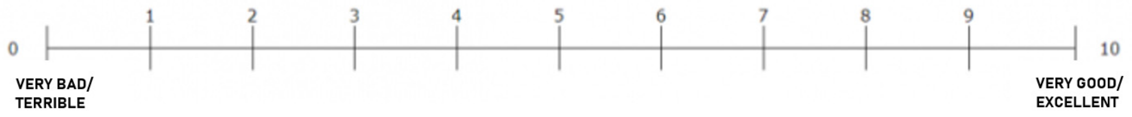

Supplement: Supplementary file 1 [file ijerph-18-03457-s001.pdf]
